# Alzheimer’s Disease and Impaired Bone Microarchitecture, Regeneration and Potential Genetic Links

**DOI:** 10.3390/life13020373

**Published:** 2023-01-29

**Authors:** Min Zhang, Shunze Hu, Xuying Sun

**Affiliations:** 1 Department of Orthopedic Surgery, Tongji Hospital, Tongji Medical College, Huazhong University of Science and Technology, Wuhan 430030, China; 2 Department of Nursing, Tongji Hospital, Tongji Medical College, Huazhong University of Science and Technology, Wuhan 430030, China; 3 Department of Pathology, Maternal and Child Health Hospital of Hubei Province, Tongji Medical College, Huazhong University of Science and Technology, Wuhan 430070, China

**Keywords:** Alzheimer’s disease, osteoporosis, genetic factors, signaling pathways, neurotransmission, therapeutic approaches

## Abstract

Alzheimer’s Disease (AD) and osteoporosis are both age-related degenerative diseases. Many studies indicate that these two diseases share common pathogenesis mechanisms. In this review, the osteoporotic phenotype of AD mouse models was discussed, and shared mechanisms such as hormonal imbalance, genetic factors, similar signaling pathways and impaired neurotransmitters were identified. Moreover, the review provides recent data associated with these two diseases. Furthermore, potential therapeutic approaches targeting both diseases were discussed. Thus, we proposed that preventing bone loss should be one of the most important treatment goals in patients with AD; treatment targeting brain disorders is also beneficial for osteoporosis.

## 1. Introduction

The central nervous system (CNS) has long been regarded as a principal manager of many physiological functions. Brain dysfunction contributes to diverse peripheral disorders. AD is one of the most common neurodegenerative diseases that mainly affects the CNS, with notable synaptic transmission impairment and cognitive decline. AD has common comorbidity and shared pathophysiology with other age-related diseases [1]. In clinical observations, patients with AD often have osteoporosis, leading to an increased incidence of bone fracture [2,3]. A study of osteoporotic subjects between 60 and 75 years old found that women had a 2.15-fold and men had a 2-fold increased risk of AD [4]. These two diseases are age-related and strongly correlated with clinical epidemiology [5,6]. Clinically, bone metabolic biomarkers and bone mineral density (BMD) are closely associated with early-stage AD patients [7]. Moreover, AD and osteoporosis are both multifactorial and polygenetic diseases, involving many similar susceptible genes. A recent study found that the most representative AD model—familial Alzheimer’s disease (5XFAD) mice overexpressing five FAD mutations—exhibited a remarkable deterioration in bone quality [8], suggesting a distinct connection between these two diseases in AD mouse models. However, there is still no clear pathological association between AD and bone.

Bone tissue is continuously remodeled, including bone resorption by osteoclasts (OCs) and bone formation by osteoblasts (OBs). It remains unknown whether AD symptoms occur first, or abnormal osteoclastogenesis and osteogenesis, or whether both occur simultaneously. However, many studies have indicated that the treatment of one disease may be beneficial for the other [9,10], although most studies have mainly focused on neural symptoms. One study showed that the neural circuit in the ventromedial hypothalamus (VMH) mediates chronic stress-induced bone loss, while optogenetic operation on this circuit rescued both anxiety-like behavior and bone loss, implying that brain therapeutic approaches can also reverse osteoporosis symptoms [11]. However, bone loss is a risk factor of cognitive decline [12,13]. Hormone replacement therapy was considered to protect against osteoporosis and AD [14]. An acetylcholinesterase inhibitor used to treat mild to moderate AD was reported to increase bone quality and promote fracture healing [15,16,17]. All these studies highlighted that AD and osteoporosis share common mechanisms. In this review, the shared mechanism and related treatment between AD and osteoporosis are discussed. The purpose of the review is to provide updated knowledge regarding the connection between and treatment of these two diseases.

To find relevant articles dated through December 2022, a systematic search was performed in PubMed using the following combination of keywords: “Alzheimer’s Disease” or “brain”, “bone”, and “mice models”, with the following enabled parameters: English language, articles published from 1985 to the present. Duplicate references were excluded. In a second step, the abstracts and methodologies of the studies were screened.

## 2. Osteoporotic Phenotype in AD Animal Models

The pathogenic factors of AD mainly include the formation of intracellular neurofibrillary tangles (NFTs), tau lesions and extracellular senile plaques (SP) development due to Aβ deposition [18,19,20]. SP and NFTs regularly appear in the neocortex, hippocampus, amygdala and basal nucleus of the brain in AD [21]. The 5XFAD mice mentioned above are an AD mouse model with both SP and NFTs pathologies in the brain, who demonstrated an obvious osteoporotic phenotype. Recently, two studies from the same group identified that early-onset osteoporosis in a tauopathy model (htau) showed alterations in Wnt/β-catenin signaling genes in both the brain and in bone [22,23]. Interestingly, most tau proteins localize on the neurons of the brain, although how tauopathy in the brain plays a role in bone quality is not clear. The above authors found overall reduced tryptophan hydroxylase (TPH) proteins in htau brainstem and a 70% reduction in TPH-positive cells in the dorsal raphe nucleus (DRN)—a pivotal structure in the regulation of the adult skeleton. Pathological changes in tau phosphorylation that occur in the serotonin-producing neurons of the brainstem raphe may have contributed to the bone loss in this mouse. However, few studies have elucidated the neural circuit changes in the hypothalamus in AD, which has been shown to be the most important brain region in the regulation of bone mass [11,24]. Additional investigations are needed to explore hypothalamus neural circuits in AD in the future.

The other hallmark of AD-SP is derived from the proteolytic processing of amyloid precursor protein (APP) by β- and γ-secretases [25]. APP is a transmembrane protein and, unlike tau proteins, APP is ubiquitously expressed in bone marrow stromal cells (BMSCs), OBs, macrophages (BMMs) and OCs. In Tg2576, an AD transgenic mouse model that ubiquitously expresses Swedish mutant APP (APPswe) under the control of a prion promoter, age-related bone loss was found with aberrant osteoclastogenesis in OCs [26] and impaired osteogenesis in BMSCs [27]. This is direct evidence that the AD pathogenic factor affects bone homeostasis. Furthermore, researchers found that serum levels of Hepcidin levels increased in Tg2576 mice; thus, the increased bone resorption observed in Tg2576 may be dependent on hepcidin upregulation [28].

The hyper-inflammation and high levels of oxidative stress observed in the brains of AD mouse models may account for the decreased bone quality in AD mice [29]. SP and NFTs deposited in the brain induce glias migration and lead to the release of increased inflammatory cytokines [30]. Cytokines such as TNF-α and IL-1β, which are released from the brain, could directly target OC and thus enhance osteoclastogenesis and hamper OB differentiation [31]. Besides, the receptor for advanced glycation end products (RAGE) is a receptor of the immunoglobulin super family, which plays various important roles under physiological and pathological conditions. RAGE acts as both an inflammatory intermediary and a critical mediator of oxidative stress. RAGE-induced AD-like pathophysiological changes drive the AD process. Cui and colleagues found that the RAGE receptor is also expressed in OCs, which mediates Aβ-induced bone resorption in Tg2576 mice [26]. The systemic inflammatory changes originate from brain, and downstream receptor alterations may account for the brain–bone axis regulation of bone mass. Despite these systemic inflammatory changes in AD models (Table 1), hormone dysfunction and common pathology factors for both AD and osteoporosis are summarized in this review.

## 3. Hormone Alterations in AD and Osteoporosis

Dysfunctions in several hormones are relevant to both AD and osteoporosis, and contribute to the development of these two diseases. Some of these hormones are given below.

FSH–Estrogen axis: Similar to the high incidence of osteoporosis observed in post-menopausal women, the female sex was more susceptible to developing AD during the pre-, peri-, or menopause period [32]. Although the pathological manifestations regarding hippocampal atrophy, brain hypometabolism, and cortical Aβ deposition are similar to those of men [33,34], women show a more rapid decline across a wide range of cognitive abilities after being diagnosed with AD [35,36]. AD inequitably affects men and women; according to the recorded incidence of AD at present [37,38], it has been estimated that almost 66% of AD patients are women [39]. The function of estrogen is generally regulated by the nuclear receptors, estrogen receptor alpha (ERα) and estrogen receptor beta (ERβ) [40,41], as well as the newly defined G protein-bound estrogen receptor-1 (GPER-1) [42]. The application of estrogen reduced brain tau hyperphosphorylation [43,44] and abolished Aβ mediated neurotoxicity in the brain [45,46]. Targeting estrogen receptors is proven to promote neural regeneration and serve as a treatment for AD [47,48]. The role of estrogen in bone remodeling is also well-elucidated; estrogen deficiency at menopause contributes to the development of osteoporosis [49]. Ovariectomy (OVX) surgery has long been regarded as a model for the induction of osteoporosis [17,50]. Follicle-Stimulating Hormone (FSH) promotes the production and secretion of estrogen, which, in turn, stimulates the growth and maturation of ovarian follicles [51]. High serum FSH was often correlated with menopausal bone loss [52,53]. A novel study found that FSH blockade improves cognitive function in mice with AD, although the bone phenotype was not investigated [54], indicating that the FSH–estrogen axis is vital in the pathogenesis of both AD and osteoporosis.

Insulin is an anabolic hormone in peripheral tissues, which plays a variety of roles. It regulates glucose metabolism, stimulates glucose transport into cells and suppresses hepatic glucose production. Insulin resistance has been recognized as the main risk factor for diabetes. Studies of epidemiology have clearly demonstrated that patients with type 1 diabetes have low BMD and are at increased risk of fractures as compared with healthy controls [55]. Bone is an insulin-responsive organ that participates in the whole-body energy metabolism [56]. Both OC and OB express the insulin receptor on their surface. Insulin stimulates OB proliferation and differentiation and has been proposed to be an anabolic agent in bone [57], although insulin is also necessary for osteoclatogenesis [58]. In vitro and animal studies indicated that insulin resistance contribute to the pathogenesis of AD through multiple different pathways [59], leading to the accumulation of SP and NFTs deposits in AD brains via inflammatory factors, mitochondrial dysfunction, oxidative stress, apoptosis, excitotoxicity and overactivation of protein kinases. Moreover, the dysregulation of insulin renders aberrant glucose and lipid levels in AD brains [60], where excessive glucose and lipid subsequently induce a secondary bone mass deterioration.

Previous studies have found that thyroid hormone (TH) levels are positively associated with brain Aβ deposition in both mice models [61,62] and postmortem human brain tissues [63,64]. Thyroid dysfunction directly leads to cognitive impairment, as observed in AD patients. Nevertheless, several studies suggested that altered TH levels are a consequence of AD. This hypothesis is explained by the secondary effects of the brain degeneration characteristic of AD; for example, adenohypophysis deterioration could lead to a decrease in thyroid-stimulating hormone (TSH) production, resulting in low TH levels [65]. TH is essential for normal bone growth and development [66]. Thus, it is worth noting that AD may decrease bone quality by reducing TH production.

Chronic psychosocial stress is increasingly being recognized as a risk factor for sporadic AD [67]. The hypothalamic–pituitary–adrenal axis (HPA axis) is the major stress response pathway in the body and tightly regulates the production of cortisol [68], a glucocorticoid hormone. Dysregulation of the HPA axis and increased levels of cortisol are commonly found in AD patients and a major contributing factor to disease progression [69,70,71]. Glucocorticoids also have a marked effect on bone metabolism, using several pathways. In bone tissues, glucocorticoids reduce osteogenesis from BMSCs and direct their differentiation to adipocytes. In addition, glucocorticoids reduce the maturation, lifespan, and function of OBs, which eventually leads to bone loss [72]. The effect of glucocorticoids on OCs is, however, controversial. Further studies found that early glucocorticoid treatment caused a transient increase in osteoclastogenesis [72], while long-term glucocorticoid treatment induced the suppression of bone resorption [73].

Melatonin is a hormone related to sleep disturbances; it is synthesized by the pineal gland. Accumulating studies have proposed that melatonin has therapeutic effects in AD treatment. For example, melatonin was reported to ameliorate cognitive deficits [74], mediate Aβ production and clearance [75], and attenuate tau aggregation [76] in AD. Decreased melatonin levels were observed in AD patients [77]. Many studies have also confirmed the beneficial effects of melatonin on bone metabolism, including enhancing bone formation [78,79] and reducing bone resorption in the bone [80,81]. Interestingly, melatonin has a 24 h circadian rhythm; some studies indicated that bone turnover also exhibits a circadian rhythm with an increase in bone resorption during thenight [82,83]. Circadian rhythm genes, especially brain and muscle ARNT-like protein-1 (BMAL1), are also a risk factor for both AD [81] and osteoporosis [84] (Table 2).

## 4. Potential Common Genetic Factors in AD and Osteoporosis

The effect of genetic factors on the incidence of AD and osteoporosis has also been investigated. Multiple risk genes/loci identified in AD patients encode proteins that are critical for bone homeostasis, such as Apolipoprotein E4 (ApoE4), triggering receptor expressed on myeloid cells 2 (TREM2), Siglec-3(CD33), proline-rich tyrosine kinase 2(PYK2), vacuolar sorting protein 35(VPS35), and sortilin-related receptor 1(SorL1) [85,86,87]. ApoE4 is the most fully investigated. ApoE4 is related to high serum cholesterol concentration [88], other lipid disturbances [89] and ischaemic heart disease [90], and has been recognized as the strongest genetic risk factor for AD. Existing data demonstrate its role in both neurons and glias [91,92,93]. One study showed, in mice lacking ApoE, increased bone formation, which was attributed to the decreased uptake of triglyceride-rich lipoproteins by the OBs [94], indicating that ApoE is also a risk factor for osteoporosis. Other studies suggest that ApoE is associated with fractures [95,96]. However, a meta-analysis involving 17 clinical reports suggested that the evidence supporting a strong and consistent association between the ApoE genotype and BMD and fracture incidence is insufficient [97], which makes the connection controversial.

TREM2 is a molecule that is highly expressed in microglia. The R47H variant of the TREM2 gene was identified to significantly increase the risk of developing AD by genome-wide associations studies (GWAS) [98]. A recent study demonstrated hemizygous for the TREM2 R47H variant (TREM2R47H/+) that does not exhibit AD pathology, and showed significant bone loss. Importantly, the bone phenotype was independent of brain phenotypes, indicating that TREM2 is also a genetic risk factor for osteoporosis [99]. Siglec-3 is a transmembrane sialic acid-binding receptor on the surface of microglial cells. GWS studies also implicated a polymorphism near CD33 as a genetic risk factor for AD [100]. Similar to microglia, OC is also derived from macrophages, while Siglec-3 is expressed on OCs. The role of Siglec-3 in bone resorption was not fully investigated, but as it occurs upstream of TREM, it is also a potential risk factor for both AD and osteoporosis.

The intracellular tyrosine kinase Pyk2 is a focal adhesion kinase and localizes to postsynaptic sites in the brain. Genetic variation in Pyk2 contributes to the late onset of AD [101]. Pyk2 plays variety of roles in bone turnover, including OBs and OCs. The deletion of Pyk2 enhances bone mass [102], suggesting another overlapping gene risk factor for AD and osteoporosis. VPS35 is a major component of the retromer complex, which is important for the endosome-to-Golgi retrieval of membrane proteins. It is identified as a potential pathogenic genes associated with osteoporosis [103]. There is evidence indicating that the VPS35 protein is also involved in the neuropathology of AD [104]. Although multiple genetic factors with concurrent AD and osteoporosis were studied, further evidence of the bidirectional molecular interaction of these two diseases is still needed (Table 3).

## 5. Similar Signaling Pathways Involved in AD and Osteoporosis

Many signaling pathways are affected in both AD and osteoporosis. The consistency pathway alterations in brain and bone tissue make it possible that the phenotype in the brain further exaggerates osteoporosis in the bone tissues. This connection allows for combined therapeutic treatment for both AD and osteoporosis. Some of the reported pathways are described in the following.

Wnt/β-catenin signaling is widely discussed in AD pathogenesis. This regulates cell proliferation, migration and differentiation. Dysfunction in Wnt/β–catenin was shown to be vital in AD progression. Wnt/β–catenin signaling promotes neuronal survival and neurogenesis, enhances synaptic plasticity, and is essential to the integrity and function of the blood–brain barrier (BBB). The activation of Wnt/β-catenin signaling suppresses tau phosphorylation, ameliorates neural inflammation and inhibits Aβ production [105]. Targeting Wnt/β-catenin is a promising approach in AD treatment. Wnt/β-catenin is also vital to bone homeostasis. For example, the conditional deletion of Ctnnb1 (which encodes β-catenin) in OBs or osteocytes resulted in severely low bone mass, while the conditional activation of β-catenin led to a dramatically increased bone mass [106,107]. Sclerostin, a glycoprotein that interrupts the Wnt/β–catenin pathway, was used as a therapeutic approach to osteoporosis treatment [108]. The tauopathy model of htau mice showed alterations in Wnt/β-catenin signaling genes in both brain and bone tissues suggestive of the increased inhibition of this pathway in an AD mice model [22]. The above studies establish a key role for Wnt/β–catenin in the brain, and in establishing and maintaining human bone mass.

Regarding bone, transforming growth factor-beta (TGF-β) and bone morphogenic protein (BMP) signaling are vital in both embryonic skeletal development and postnatal bone homeostasis [109]. BMP is a member of the TGF-β subfamily, which has important effects on neuronal differentiation and axonal growth. The BMPs have more than 20 isoforms. They are first discovered in bone tissue and activate the canonical small mother against decapentaplegic (Smad) pathway in the brain via their type I and type II Serine/Threonine kinase receptors. Studies have identified BMPs in mice as promoting bone regeneration and the rehabilitation of critical-size bone defects, which renders them useful in the field of tissue engineering and regeneration. Smad4 is the most common Smad for both TGF-β and BMP signaling [110]. The deletion of Smad4 in mice results in numerous developmental defects and cancer formation in various tissues. Studies revealed that Smad4 inhibited bone resorption and promoted osteogenesis in bones. Interestingly, TGF-β/BMP signaling was also essential in brain development, while altered TGF-β expression was observed in AD brain and cerebrospinal fluid. Targeting TGF-β/BMP renders restored cognitive decline in AD. TGF-β was shown to restore hippocampal synaptic plasticity and memory in AD mice models [111]. The conditional deletion of Smad4 in adult neural stem cells severely impairs neurogenesis [112]. These findings illustrate that the TGF-β/BMP is a common pathway, involved in both AD and osteoporosis.

Hippo/Yes-associated protein 1(YAP1) signaling has recently attracted attention in the field of AD and osteoporosist. YAP/TAZ-TEAD complexes activate the expression of many target genes, and are thus able to regulate a variety of cellular processes. YAP is an important regulator of proliferation and cell cycle during mammalian neurogenesis [113]. The activation of YAP signaling partially rescued the senescence of astrocytes and improved the cognitive function of AD model mice and aging mice [114]. In bone tissues, YAP was reported to promote bone development [115]. Furthermore, YAP promotes osteogenesis and suppresses adipogenic differentiation in OBs [116], as well as inhibiting bone resorption [117] (Table 4).

## 6. Impaired Neurotransmitter in AD Brain

AD is characterized by degenerative changes in a variety of neurotransmitter systems. The most pronounced neurochemical abnormality in AD is the loss of cholinergic tone in the central nervous system [118]. AD treatment has been dominated by the use of acetylcholinesterase (AChE) inhibitors. Cholinergic fibers innervate bone and transmit anabolic signals from the brain and mediate bone resorption in OCs [119]. A cholinergic neuroskeletal interface was demonstrated to promote bone formation through bone-anabolic effects [120], indicating neural regulation in the brain–bone axis.

Dopamine represents another important molecule; decreased dopamine levels were found in the AD brain [121]. Dopamine receptors were reported to suppress bone resorptions, further suggesting that brain function affects bone homeostasis [122].

Glutamate is an excitatory transmitter during synaptic activities. High levels of glutamate in AD patients are often related to aberrant extrasynaptic N-methyl-D-aspartate (NMDA) receptor overactivation, which, in turn, causes high incidence of epilepsy in the brain. Extrasynaptic NMDA receptor activation markedly increases Aβ production [123]. Our previous findings suggested that excessive glutamate release caused by the overactivation of extrasynaptic NMDA receptor results in tau hyperphosphorylation in the brain [124], indicating the neurotoxicity of glutamate in the brain. Although it is unclear whether OBs or OCs express NMDA receptors, the glutamate/aspartate transporter (GLAST) is expressed by OB and embedded osteocytes. Evidence has shown that glutamate reduces bone formation, while it promotes bone resorption [125] in bone tissues.

Another brain-derived peptide-neuropeptide Y (NPY) is a highly conserved 36-amino-acid peptide that is usually abundant in the CNS. This is a major regulator of food consumption and energy homeostasis, which can also efficiently promote neurogenesis and inhibit inflammation in AD brain [126]. In the periphery tissues, NPY can be released from the sympathetic nerves and adrenal medulla [127]. Mice lacking osteocyte NPY exhibit a high bone mass phenotype and significant reduction in aging- and OVX-associated bone loss and marrow fat accumulation [128]. These studies recapitulate that nerve fibers deriving from the central nervous system may directly regulate skeletal bone development, furthering the evidence that brain activity controls bone remodeling.

Cocaine amphetamine regulated transcript (CART), an anorexic neuropeptide precursor protein, is also involved in the regulation of food intake and energy expenditure. It is released in the ventral tegmental area of the brain according to the serum levels of leptin [129]. Low hypothalamic CART levels were associated with increased bone resorption with higher levels of RANKL; thus, elevated CART rescues the osteoporosis phenotype. CART treatment consistently improves AD cognitive decline by mitigating oxidative stress and DNA damage in animal models [130,131]. All these neural factors support the idea that altered brain neuropeptides, induced by dysfunctions in brain neurotransmission, would likely lead to abnormal bone homeostasis (Table 5).

## 7. Therapeutic Strategies Targeting AD and Osteoporosis

Peripheral tissue therapies to rescue brain disorders have been suggested by many investigations. Brain-derived neurotrophic factor (BDNF), which has been shown to be a mediator of activity-induced LTP in the hippocampus, as well as in other brain regions [132,133], was able to ameliorate cognitive loss in AD mice models [134,135]. The application of BDNF revealed enhanced bone formation and fracture healing [136,137]. Its receptor, tropomyosin-related kinase B receptor TrkB, was also reported to prevent bone loss [138] and enhance fracture-healing [139]. These findings provided the possibility of combined treatment for both AD and osteoporosis. The bone-derived hormone osteocalcin (OCN) is also crucial to brain development and neural cognitive functions, whose absence led to decreased bone quality in mice [140,141]. It can cross the blood–brain barrier and bind to neurons of the brainstem, midbrain, and hippocampus, as well as influencing the synthesis of several neurotransmitters [142,143]. The deletion of OCN can cause abnormal embryonic brain development and age-related cognitive decline [144]; this may explain why improved bone quality could attenuate cognitive loss in the AD brain.

In addition to these traditional cholinesterase inhibitors and NMDA receptor antagonist, which inhibit neurodegeneration or activate neural regeneration or clear the Aβ deposits when treating AD, here we will discuss stem cell therapies and tissue engineering applications in AD treatment and identify whether these treatments are also beneficial in osteoporosis.

Stem cell therapy has powerful potential for the treatment of AD. Many stem cell sources have been used to treat AD, such as neural stem cells (NSCs), embryonic stem cells (ESCs), and mesenchymal stem cells (MSCs) from bone marrow, umbilical cord and umbilical cord blood. Patient-specific-induced pluripotent stem cells (iPS cells) are proposed as a future prospect in the treatment of AD in animal models [145]. The above-mentioned stem cells are all multi-potent; there can be little doubt that stem cell therapies are beneficial to osteoporosis. The most popular stem cell source is MSC. Preclinical investigations on MSC transplantation proved that MSC enhanced osteogenic differentiation, increased bone mineral density, and halted the progression of osteoporosis. The feasibility of adipose-tissue-derived mesenchymal stem cell (ATMSC)-based treatment for osteoporosis was also investigated [146]. Furthermore, umbilical cord blood has recently become an alternative stem cell source for osteoporosis treatment [147]. Moreover, the latest techniques, such as gene modification, targeted modification and co-transplantation, are promising approaches to enhance the therapeutic effect and efficacy of MSCs. Clinical trials focusing on the use of MSC therapy to treat AD and osteoporosis are underway [148]. Although a high number of studies have confirmed their efficiency in treating AD and proved that those stem cells can survive and maturate to functional neural cells in animal models of AD, their clinical application still needs further investigation.

Recently, extracellular vesicles (EVs) have attracted considerable attention regarding the interaction of different cells. AD brain-derived EVs have been shown to contain many pathogenic proteins, such as Aβ, hyper-phosphorylated tau and α-synuclein [149,150], and EVs were then secreted into biofluids such as blood, urine and CSF [151,152], and circulated throughotu the entire body. It is unclear whether these toxic EVs could target bone cells, since few studies have investigated this. However, EVs from peripheral tissues were reported to have the ability to attenuate the cognitive loss of AD. EVs from BMSCs were verified to be able to promote osteogenesis and render bone regeneration. 5XFAD mice that received human MSC-EVs treatment behaved significantly better in cognitive tests than saline-treated 5XFAD mice [153]. Another study revealed that young osteocytes, the most abundant cells in bone, secrete EVs (OCY^Young^-EVs) to ameliorate cognitive impairment and the pathogenesis of AD in APP/PS1 mice model and cells [154]. Similarly, lateral ventricle administration, but not caudal vein injection of BMSC-EVs, improves AD-like behavioral performance in STZ-injected mice; this mechanism might be involved in the regulation of glial activation and its associated neuroinflammation and BDNF-related neuropathological changes in the hippocampus [155]. The above findings indicated that peripheral bone-marrow-deprived EVs have a therapeutic effect on AD treatment. Although there is still a long way to go before the clinical application of peripheral EVs, these findings are encouraging for the potential use of peripheral regenerative factors in AD treatment.

## 8. Conclusions

**Figure 1:**
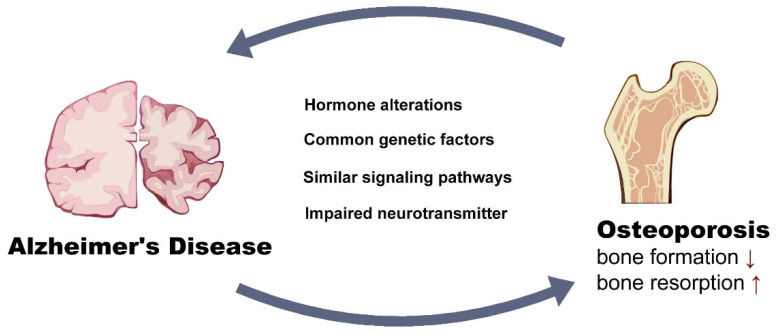
Potential links that worsen both Alzheimer’s Disease and osteoporosis.

The correlation between AD and osteoporosis has been of great interest in the field of the brain–bone axis. The understanding of the connection of these two diseases is summarized in Figure 1. Although the exact signaling pathways in this axis are diverse, they are inseparably linked with each other. There are still many unknown points regarding the understanding of the pathology of these two diseases. For example, the brain–gut axis has increasingly been noticed as regulating inflammation [156] and immune response [157]. Reduced bone quality is associated with an altered microbiota; thus, whether AD orchestrates bone loss through microbiota needs to be further explored. Moreover, the brain circuit was proven to directly regulate peripheral immune system. It is no surprise that brain circuit changes in AD may directly regulate the immuno-response in bone tissues and, in turn, affect bone quality, but how this was achieved in the AD brain circuit was elusive. The significantly lower vitamin D levels in patients with dementia compared to cognitively intact controls also contributes to the low bone mass in AD patients [158]. Reduced exposure to sunlight in patients with AD has been implicated as the main cause of vitamin D deficiency in patients with dementia, conferring an environmental risk factor in the common pathogenesis of these two diseases [159]. Understanding the common pathogenesis of AD and osteoporosis may lead to more effective therapeutic strategies for treating the dual diseases.

## 9. Future Directions

Overall, this review points to the importance of further studies on the association between bone loss in AD patients and suggests that more attention should be paid to the development of new treatments for these two diseases. Bone loss prevention not only decreases the economic burden on society, but also improves cognitive levels in AD patients. Reducing bone loss and fracture should be one of the most important treatment goals in patients with AD.

## Figures and Tables

**Table 1:** Osteoporotic phenotype in AD animal models.

AD Mice Model	Authors	Reference Number
5XFAD	JE et al.	[8]
Htau	Dengler et al.	[22,23]
Tg2576	Cui et al. Xia et al.	[26,27]

**Table 2:** Hormone alterations in AD and osteoporosis.

Altered Hormones	Authors	Reference Number
FSH–Estrogen	Uddin et al. Cheung et al. Wang et al.	[32,52,53]
Insulin	Hofbaner et al. Yang et al. Kim et al.	[55,57,58]
TH	O’barr et al. Ghenimi et al. De et al.	[61,62,66]
HPA axis	Hdsboer et al. Weinstein et al. Teitelbaum et al.	[68,72,73]
Melatonin	Hardeland et al. Zhang et al. Park et al.	[77,78,79]

**Table 3:** Common genetic factors in AD and osteoporosis.

Genetic Factors	Authors	Reference Number
ApoE4	Schiling et al. Zajickova et al. Huang et al.	[94,95,96]
TREM2	Korvatska et al.	[98]
Siglec-3	Estns et al.	[100]
Pyk2	Lee et al. Sun et al.	[101,102]
VPS35	Xia et al. Wen et al.	[103,104]

**Table 4:** Signaling pathways involved in AD and osteoporosis.

Signaling Pathways	Authors	Reference Number
Wnt/β-catenin	Jia et al. Glass et al. Kramer et al.	[105,106,107]
TGF-β	Wu et al. Hu et al. Colak et al.	[109,111,112]
YAP1	Xu et al. Pan et al. Yang et al.	[114,116,117]

**Table 5:** Neurotransmitters in AD brain that regulate bone homeostasis.

Genetic Factors	Authors	Reference Number
AChE	Bajayo et al. Gadomski et al.	[119,120]
Dopamine	Chen et al. Hanami et al.	[121,122]
Glutamate	Sun et al. Skerry et al.	[124,125]
NPY	Duarte et al. Zhang et al.	[126,128]
CART	Singh et al. Jin et al. Belelovsky et al.	[129,130,131]

## Data Availability

No new data were created or analyzed in this study. Data-sharing is not applicable to this article.
